# Proteomic insights into biology of bipolar disorder: implications for health complexity and mortality

**DOI:** 10.47626/2237-6089-2024-0820

**Published:** 2025-04-17

**Authors:** Paola Rampelotto Ziani, Marco Antônio de Bastiani, Pietra Paiva Alves, Pedro Henrique da Rosa, Tainá Schons, Giovana Mezzomo, Ellen Scotton, Flávio Kapczinski, Adriane R. Rosa

**Affiliations:** 1 Hospital de Clínicas de Porto Alegre Laboratório de Psiquiatria Molecular Porto Alegre RS Brazil Laboratório de Psiquiatria Molecular, Hospital de Clínicas de Porto Alegre (HCPA), Porto Alegre, RS, Brazil.; 2 Universidade Federal do Rio Grande do Sul Departamento de Farmacologia e Instituto de Ciências Básicas da Saúde Programa de Pós-Graduação em Farmacologia e Terapêutica Porto Alegre RS Brazil Programa de Pós-Graduação em Farmacologia e Terapêutica, Departamento de Farmacologia e Instituto de Ciências Básicas da Saúde, Universidade Federal do Rio Grande do Sul (UFRGS), Porto Alegre, RS, Brazil.; 3 UFRGS Faculdade de Medicina Programa de Pós-Graduação em Psiquiatria e Ciências do Comportamento Porto Alegre RS Brazil Programa de Pós-Graduação em Psiquiatria e Ciências do Comportamento, Faculdade de Medicina, UFRGS, Porto Alegre, RS, Brazil.

**Keywords:** Mass spectrometry, molecular signatures, bioinformatics, biomarkers, complement system, coagulation cascade

## Abstract

**Objective:**

The present study has the following objectives: 1) identify differentially expressed proteins and pathways in blood samples of bipolar disorder (BD) compared to healthy controls (HC) by employing high-throughput proteomics and bioinformatics; and 2) characterize disease-related molecular signatures through in-depth analysis of the differentially expressed proteins and pathways.

**Methods:**

Blood samples from patients with BD (n = 10) classified into high (BD+) or poor functioning (BD-), based on functional and cognitive status, and HC (n = 5) were analyzed using mass spectrometry-based proteomic analysis. Bioinformatics was performed to detect biological processes, pathways, and diseases related to BD.

**Results:**

Eight proteins exclusively characterized the molecular profile of patients with BD+ compared to HC, while 26 altered proteins were observed in the BD- group. These altered proteins were mainly enriched in biological processes related to lipid metabolism, complement system and coagulation cascade, and cardiovascular diseases; all these changes were more prominent in the BD- group.

**Conclusion:**

These findings may represent systemic alterations that occur with the progression of the illness and a possible link between BD and medical comorbidities. Such comprehensive understanding provides valuable insights for targeted interventions, addressing mental and physical health aspects in subjects with BD. Despite these promising findings, further research is warranted, encompassing larger sample cohorts and incorporating biological validation through molecular biology methods.

## Introduction

Bipolar disorder (BD) is a highly disabling disease associated with elevated rates of premature mortality.^1,2^ Patients with BD died 9 years younger than the general population.^3^ This occurs not only by suicide but also due to a high prevalence of medical comorbidities.^4,5^ A recent meta-analysis of mortality in subjects with BD showed that they have a twofold increased risk of dying prematurely due to medical diseases when compared to the general population.^6^ The Task Force of the International Society for Bipolar Disorders (ISBD) also suggested a strong association between BD and cardiovascular disease, raising questions regarding the reasons for this link. Unhealthy lifestyle factors may contribute to the high rates of medical comorbidities in BD.^5^ In addition to concerns about physical well-being, these risk factors lead to more severe manifestations of BD. For example, individuals with both BD and obesity are at a higher risk of encountering more severe mood symptoms, increased resistance to treatment, and cognitive and functional impairment.

Multifactorial models, including inflammation, oxidative stress, mitochondrial dysfunction, and neurotrophic factors, partially explain BD's accelerated aging and premature mortality.^7-9^ Despite these known mechanisms, our understanding of the systemic changes observed throughout the BD remains incomplete. The existing literature has limitations, such as focusing on isolated mechanisms, emphasis on behavioral factors, and limited approaches. There is a tendency to analyze factors individually without considering the complex interaction between different molecular pathways that contribute to the pathophysiology of BD. The influence of unhealthy lifestyle factors on medical comorbidities of BD is frequently studied, but this does not fully explain the relationship between the disease and early mortality, and current methodologies may not capture the full spectrum of molecular changes associated with BD. To overcome these limitations, we need new approaches that analyze the interplay between multiple molecular pathways and search underlying biological markers that help explain the relationship between BD and medical diseases.

In this sense, to address the limitations of current understanding and explore the multifaceted molecular mechanisms underlying BD and its associated medical comorbidities, the present study has two key objectives: 1) identify differentially expressed proteins and pathways in blood samples of BD compared to healthy controls (HC) by employing high-throughput proteomics and bioinformatics; and 2) characterize disease-related molecular signatures through in-depth analysis of the differentially expressed proteins and pathways.

## Methods

### Subjects

Ten female participants were recruited by convenience from Programa de Tratamento do Transtorno de Humor Bipolar (PROTHABI) at Hospital de Clínicas de Porto Alegre (HCPA). The inclusion criteria were: (1) diagnosis of BD according to Diagnostic and Statistical Manual of Mental Disorders, 5th edition (DSM-5) (Structured Clinical Interview for DSM-5 [SCID]) and (2) age between 18 and 70 years. Exclusion criteria were clinical diagnosis of intellectual disability and/or Alzheimer's disease, uncontrolled medical conditions (as determined by medical records, self-report, or routine blood tests), autoimmune diseases, cancer, substance use, alcohol abuse or dependence, and pregnancy or lactation, and electroconvulsive therapy within the past year. All patients received pharmacological treatment according to the program's protocols.

The control group comprised five healthy volunteers and blood donors from the hemocenter at HCPA. These participants had no current or previous history and no first-degree family history of a major psychiatric disorder, including dementia or intellectual disability, assessed by the non-patient version of the SCID. They also had no uncontrolled medical conditions (as determined through self-report or routine blood tests) and no autoimmune diseases. Participants were matched according to gender and age.

### Ethical considerations

Before participating in the study, all patients provided written informed consent. The study protocol was approved by the Research Ethics Committee of the HCPA (2019-0640).

### Clinical assessment

We obtained patients’ sociodemographic, clinical, and pharmacological data through a structured interview and from medical records. In particular, the clinical assessment encompassed the following scales: Montgomery-Asberg Depression Rating Scale (MADRS), Young Mania Rating Scale (YMRS), Patient-Reported Outcomes Measurement Information System for depression (PROMIS Depression), Patient-Reported Outcomes Measurement Information System for anxiety (PROMIS Anxiety), Cognitive Bipolar Rating Assessment (COBRA), and Functioning Assessment Short Test (FAST).

The FAST assessed the overall functional outcome, an instrument widely used in patients with BD. This scale encompasses 24 items evaluating six functional domains (autonomy, occupational functioning, cognitive functioning, financial issues, interpersonal relationships, and leisure time). The overall FAST score varies from 0-72 (the cut-off value is 11); the higher the score, the greater the disability.^10^ Cognitive difficulties in daily life situations were assessed with COBRA. The COBRA is a 16-item self-report instrument with satisfactory psychometric properties developed to assess cognitive complaints experienced by bipolar patients. The COBRA total score is obtained when the scores of each item are added up. The COBRA total score ranges from 0-48 (the cut-off value is 10); a higher score means a more significant dysfunction.^11,12^ For the present study, patients were also classified into high (BD+) or poor (BD-) functioning based on their functional and cognitive status, which was assessed using the FAST and COBRA.^10-13^ Therefore, patients with BD were classified into BD+ if they met FAST scores < 11 and COBRA < 10, while those with FAST scores greater than 40 and COBRA greater than 25 were classified into BD-.

### Sample collection and depletion of high abundant proteins

The blood samples (10 mL) were collected in non-anticoagulant tubes from all participants and then centrifuged at 1,500 *g* for 15 minutes at room temperature to separate the serum. The serum was aliquoted and stored at −80 °C for further analysis. The most abundant proteins were removed with the Top14 Abundant Protein Depletion Mini Spin Columns following the manufacturer's specifications (High Select™ Depletion Spin Columns, Thermo Scientific™). Each column contains 400 µL of a 50% slurry in 10 mM PBS, 0.02% sodium azide, pH 7.4, and is optimized to bind up to 10 µL of serum. This process enhances the detection of low-abundance proteins when using mass spectrometry analysis.

### Liquid chromatography-mass spectrometry/mass spectrometry (LC-MS/MS) analysis

Depleted serum was inserted into a Microcon^®^ 5 kDa centrifugal filter and washed with Tris-HCl and the retained volume. A portion corresponding to 40 µg of protein was then dried and disulfide bonds were ruptured by dithiothreitol and urea. After iodoacetamide incubation, urea dilution, and trypsin digestion, the resulting peptides were desalted using C18 Stage Tips. Identification of serum proteins was performed using a Dionex Ultimate 3000 RSLC nanoUPLC system coupled to an Orbitrap Fusion Lumos Mass Spectrometer. Peptides were separated through reversed-phase chromatography, and mass spectrometry analysis was conducted using high-resolution Orbitrap mass analyzers. Data-dependent scans facilitated the isolation and fragmentation of selected peptides, providing a detailed analysis of the serum protein composition.

### Data analysis

Demographic data and clinical characteristics were expressed as medians and interquartile ranges (IQR) (percentiles 25th and 75th), and group comparisons were conducted using the Kruskal-Wallis test with Dunn post-hoc analysis. Statistical analyses were performed using SPSS 18 for Windows, provided by SPSS Inc. in Chicago, Illinois, USA.

The peptide spectrum match (PSM) analysis was conducted using MaxQuant version 2.2.0.0, with searches performed against the UniProt Human database. Specific parameters, including trypsin-specific digestion, variable modifications such as oxidation (M) and protein N-acetylation, and fixed modifications like carbamidomethyl (C), were employed. Additional settings included a peptide tolerance of 7 ppm, MS/MS tolerance of 0.1 Da, allowance for two missed cleavages, and a 1% false discovery rate (FDR) for both PSM and protein assignment. Relative quantification of proteins was accomplished using MaxLFQ intensity values.

### Protein-protein interaction (PPI) network, biological processes, and Kyoto Encyclopedia of Genes and Genomes (KEGG) pathways

Search Tool for the Retrieval of Interacting Genes/Proteins (STRING) is a database and online resource that contains information on known and predicted PPI. The Enrichr software application^14^ was utilized to attribute functional significance to the identified proteins. The analysis focused on the differentially expressed proteins to identify biological processes and KEGG pathways associated with BD. Subsequently, the bar chart generated by Enrichr was sorted based on the ranking of combined scores. The combined score is calculated by taking the logarithm of the p-value derived from the Fisher exact test and multiplying it by the z-score, representing the deviation from the expected rank. Terms with higher combined scores are considered the most relevant in the analysis.

### Weighted correlation network analysis

In this study, we used the R software package Weighted Gene Co-expression Network Analysis (WGCNA) to perform weighted correlation network analysis aspects. WGCNA was employed to identify clusters (modules) of highly interconnected genes and to summarize these clusters using the module *eigengene*, which is considered representative of the gene expression profiles within a module. The module *eigengenes* were assessed for correlation with external phenotypes using Pearson's correlation coefficient.^15^ We utilized BD groups as the primary outcome phenotype. Then, we performed analyses to investigate whether confounding variables such as the number of medications, number of hospitalizations, number of suicide attempts, and COBRA score also exhibited significant correlations with module *eigengenes*.

## Results

The demographic and clinical characteristics of the participants are summarized in Table 1. Gender homogeneity was maintained among the groups, as all participants were women. No significant differences in age between the groups were observed (p = 0.897). As expected from the selection criteria, BD- subjects had a higher mean FAST score (p < 0.001) and COBRA score (p < 0.001) when compared to BD+ and HC. Considering the number of medications used, BD- exhibited a higher number than HC (p = 0.004).

**Table 1:** Clinical and demographic data

Variable	Controls (n = 5)	BD- (n = 5)	BD+ (n = 5)	p-value*
Age, years	47 (45-55)	54 (52-55)	57 (36-60)	0.954
Weight, kg	73.5 (63-78.5)	82 (81-103)	72 (62-75)	0.132
BMI, kg/m²	27.48 (27.18-28.72)	32.03 (32.64-41.40)	26.47 (26.49-26.77)	0.065
Number of medications	0 (0-3)^a^	5 (4-6)^b^	2 (2-3)^ab^	**0.013**
Number of hospitalizations	0 (0-0)^a^	5 (2-6)^b^	2 (1-2)^ab^	**0.005**
Number of suicide attempts	0 (0-0)^a^	2 (1.5-6)^b^	0 (0-0)^a^	**0.005**
MADRS score	2 (0-6)^ab^	27 (26-32)^a^	0 (0-1)^b^	**0.006**
YRMS score	0 (0-1)^ab^	4 (3-6)^a^	0 (0-0)^b^	**0.008**
COBRA score	12 (4-20)^ab^	29 (28-44)^a^	2 (2-3)^b^	**0.005**
FAST score	1 (1-5)^a^	49 (48-52)^b^	5 (3-5)^b^	**0.007**

BD- = bipolar disorder with poor functioning; BD+ = bipolar disorder with high functioning; BMI = body mass index; COBRA = Cognitive Bipolar Rating Assessment; FAST = Functioning Assessment Short Test; kg = kilogram; m = meter; MADRS = Montgomery-Asberg Depression Rating Scale; YMRS = Young Mania Rating Scale.

Data expressed as median and interquartile range (IQR), percentiles 25th and 75th.

Different letters indicate significant differences among groups.

Bold values indicate p < 0.05. Significance set at 5% for all analyses.

* Kruskal-Wallis test with Dunn post-hoc analysis.

**Figure 1:**
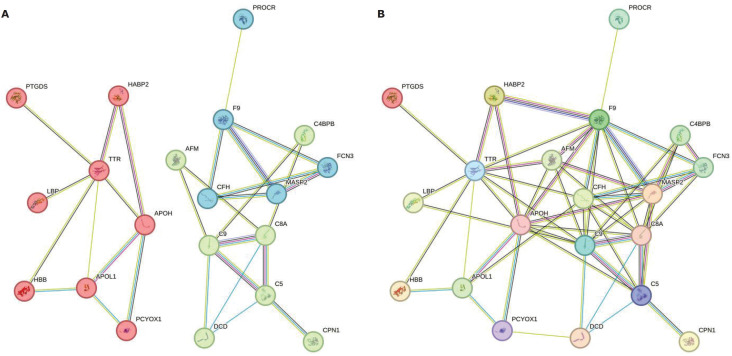
Protein-protein interaction (PPI) network. The differentially expressed proteins in the bipolar disorder (BD) vs. healthy control (HC) comparison revealed the following interaction network and their respective clusters. A) Cluster. B) PPI.

The present study was conducted to identify differentially expressed proteins between BD and HC based on LC-MS/MS technology. In total, 3506440 MS/MS spectra were obtained from 30 LC-MS/MS runs, of which 128,163 peptide sequences were identified using 1% FDR. The LC-MS/MS detected 234 proteins corresponding to the identified peptides for the 15 participants. Figure 1 presents a PPI network generated using STRING that illustrates the interactions between proteins in the BD and HC groups.

After statistical analysis, we identified 21 differentially expressed proteins in BD vs. HC, 12 in BD+ vs. HC, and 30 in BD- vs. HC, as shown in Table 2. The Venn diagram displays the intersection of proteins identified in the three comparison groups. Furthermore, four proteins – C4BPB, C5, C9, and DCD – were consistently present across all comparisons (Supplementary Figure S1 and Table S1). Most proteins identified as significantly differential in BD were involved in the lipid metabolism, complement, and coagulation cascade (e.g., apolipoproteins, complement factors, and coagulation factors).

**Table 2:** Molecular signatures from patients with BD and HC blood samples

Gene names	Protein IDs	Protein names	Log FC		
			BD/HC	BD+/HC	BD-/HC
AFM	P43652	Afamin	0.21		
APOA1	P02647	Apolipoprotein A1			0.24
APOA2	V9GYM3	Apolipoprotein A2			0.53
APOF	Q13790	Apolipoprotein F			0.59
APOH	P02749	Apolipoprotein H	0.31	0.37	
APOL1	O14791	Apolipoprotein L1	-0.61		
B2M	P61769	Beta-2-microglobulin			-0.63
C1QC	P02747	Complement C1q C chain			0.47
C3	P01024	Complement C3			-0.17
C4BPB	P20851	Complement component 4 binding protein beta	-0.36	-0.42	-0.29
C5	P01031	Complement factor C5	-0.43	-0.50	-0.36
C8A	P07357	Complement C8 alpha chain	-0.25		-0.30
C9	P02748	Complement factor C9	-0.91	-0.83	-0.99
CFD	P00746	Complement factor D		0.65	
CFH	P08603	Complement factor H	-0.35		-0.41
CPN1	P15169	Carboxypeptidase N subunit 1	0.37	0.45	
CSF1R	P07333	Colony stimulating factor 1 receptor			-0.71
DCD	P81605	Dermcidin	-1.21	-0.61	-1.8
F12	P00748	Coagulation factor XII			0.56
F9	P00740	Coagulation factor IX	-0.27		-0.38
FCN3	O75636	Ficolin 3	-0.35		-0.38
HABP2	Q14520	Hyaluronan binding protein 2	-0.21		
HBB	P68871	Hemoglobin subunit beta	-1.37	-1.98	
IGF1	P05019	Insulin-like growth factor 1			0.86
IGF2	P01344	Insulin-like growth factor 2			0.53
IGFALS	P35858	Insulin-like growth factor binding protein acid labile subunit			0.72
IGHA1	P01876	Immunoglobulin heavy constant alpha 1			0.95
IGHG2	P01859	Immunoglobulin heavy constant gamma 2			1.29
IGHG3	P01860	Immunoglobulin heavy constant gamma 3			1.90
IGLL5	P0CG04	Immunoglobulin			1.92
LBP	P18428	Lipopolysaccharide binding protein	-0.88	-1.08	
LDHB	P07195	Lactate dehydrogenase B	-0.54	-0.69	
LGALS3BP	Q08380	Galectin 3 binding protein			-1.49
MASP2	O00187	Mannan-binding lectin-associated serine protease 2	-0.92		
ORM2	P19652	Orosomucoid 2			-0.70
PCYOX1	Q9UHG3	Prenylcysteine oxidase 1	-0.66	-0.66	
PROCR	Q9UNN8	Protein C receptor	0.70		0.79
PTGDS	H0Y5A1	Prostaglandin D2 synthase	0.39		0.43
Q6NSD3	Q6NSD3	Complement factor H related 3			-0.18
SELL	P14151	Selectin L		-0.40	
THBS4	P35443	Thrombospondin 4			-0.68
TTR	P02766	Transthyretin	0.39		
VWF	P04275	Von Willebrand factor			-1.13

BD = bipolar disorder; BD- = BD with poor functioning; BD+ = BD with high functioning; HC = healthy controls.

Comparisons considering unadjusted p < 0.05 and a fold change over 15%.

**Figure 2:**
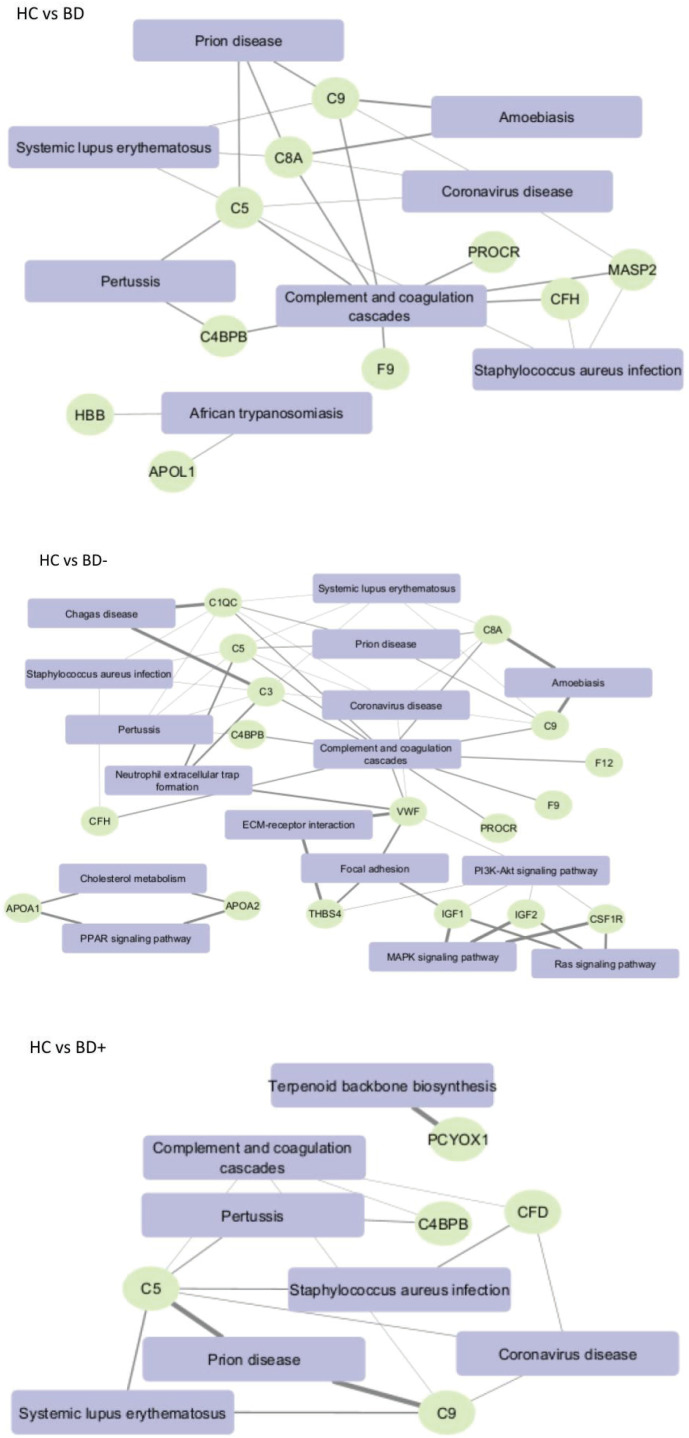
Kyoto Encyclopedia of Genes and Genomes (KEGG) pathway analysis. Complement and coagulation cascade were the two main signaling pathways involved in bipolar disorder (BD), BD with high functioning (BD+) and BD with poor functioning (BD-). We considered an adjusted p < 0.00001 computed using the Benjamin-Hochberg method. HC = healthy controls.

**Figure 3:**
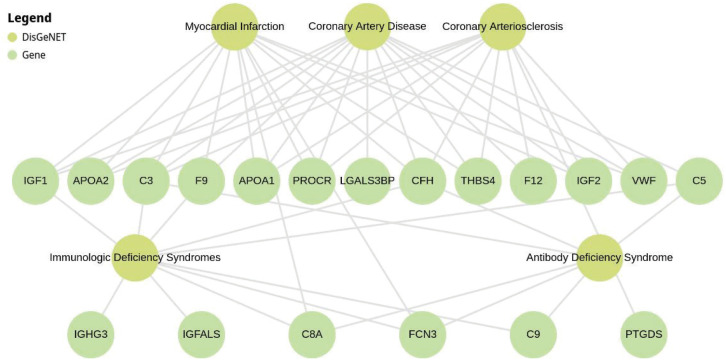
Enrichr analysis illustrating gene enrichment results using DisGeNET data. The figure highlights the association between bipolar disorder (BD) with poor functioning (BD-) genes and cardiovascular diseases. We considered an adjusted p < 0.00001 computed using the Benjamin-Hochberg method.

We utilized the Enrichr software^14^ to detect KEGG pathways and diseases associated with BD. In all comparisons, the prominent signaling pathway identified through KEGG is the complement and coagulation cascade (Figure 2). Notably, the identified proteins in the BD- group were associated with cardiovascular diseases (Figure 3).

**Figure 4:**
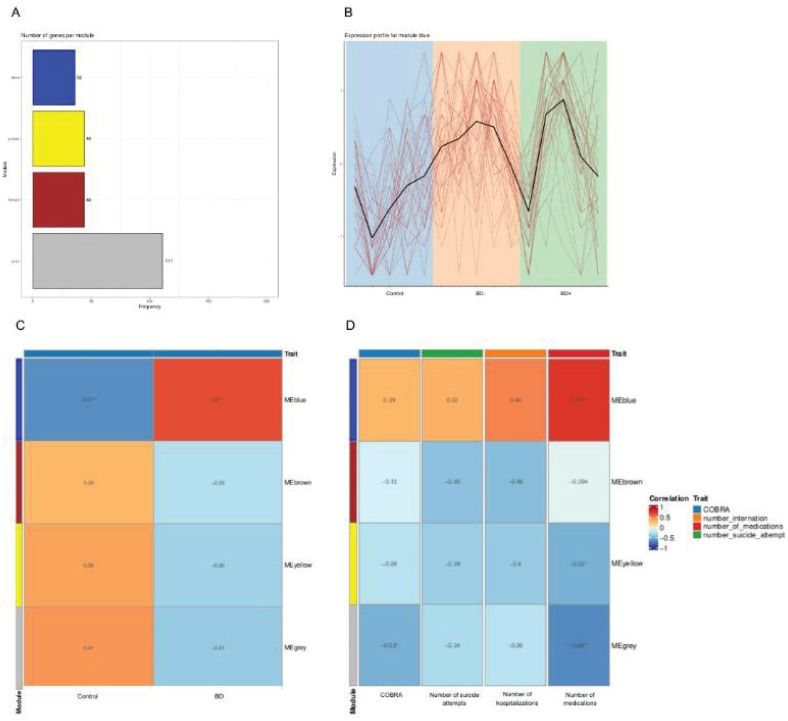
Weighted Gene Co-expression Network Analysis (WGCNA) for the blue module. A) Number of proteins per module. B) A gene expression pattern in the healthy controls (HC) group shows a negative distinction from the bipolar disorder (BD) poor functioning (BD-) and BD with high functioning (BD+) groups. C) Positive correlation between the blue module (y-axis) and the BD group (x-axis) and a negative correlation between the blue module and the HC group. The brown, yellow, and gray modules did not correlate significantly. D) The blue module exhibits a robust positive correlation with the quantity of medications our patients use. The remaining modules only display negative correlations with the covariates. COBRA = Cognitive Bipolar Rating Assessment.

WGCNA identified a co-expression network comprising four distinct modules in our sample (Figure 4A). Only one of these four modules, consisting of 36 co-expressed genes and denoted the "blue module," was significantly correlated with BD (r = 0.67). The blue module was also correlated (r = 0.74) with the number of medications used by patients, suggesting this module might be involved in biological processes related to BD and its treatment response (Figure 4D).

## Discussion

Using a combined proteomic approach and bioinformatics, the current study revealed molecular signatures and pathways correlated with BD. Eight proteins exclusively characterized the molecular profile of patients with BD+ compared to HC, while 26 proteins, that is, three times more altered proteins, were observed in the BD- group. The altered proteins were mainly enriched in biological processes related to the complement system, coagulation cascade, lipid profile, and cardiovascular diseases; all these changes were most prominent in the BD- group. Thus, this is the first study to show, through mass spectrometry-based proteomic analysis, molecular patterns, pathways, and primary medical diseases related to different stages of BD. Despite these encouraging discoveries, further research is warranted, encompassing larger sample cohorts and incorporating biological validation through molecular biology methods.

The differential proteins and biological processes identified in this study can represent systemic changes that occur with the progression of the disease or even the treatment response. The "blue module" identified by the WGCNA analysis showed a correlation between BD diagnosis and the number of medications. Polypharmacotherapy can be regarded as an indicator of disease complexity, suggesting the presence of various health conditions and a challenge in managing these conditions. We showed that medical comorbidities, in particular, cardiovascular diseases, were more preeminent in BD- suggesting this subgroup of patients may experience a more chronic course of the illness and poor prognosis. An interesting study showed that premature mortality in BD is linked to various causes, including myocardial infarction, diabetes, chronic obstructive pulmonary disease, pneumonia, and suicide.^16^ Metabolic, respiratory, and cardiovascular diseases are among the top 10 causes of death worldwide and are notably highly prevalent comorbidities in BD. Therefore, medical comorbidities in individuals with BD can exacerbate the mortality disparity between them and the general population.^17^ Many of these comorbidities are closely linked to the complement system,^18^ coagulation cascade,^19^ and lipid metabolism.^20^

Regarding biological processes and pathways, our findings support previous studies,^21-24^ indicating that the interplay between the complement system and coagulation cascade is involved in the etiology and progression of BD. The complement system originates from the serine protease reaction cascade, sharing a common ancestry with the coagulation factors encoded by the same ancestor genes.^25,26^ Beyond their common origin, these systems exhibit similar roles in promoting the initial defense against infections and tissue repair, potentially contributing to homeostasis or the onset of pathological conditions. Analogous to the complement system, the coagulation cascade is a tightly regulated and coordinated process culminating in clot formation, constituting the hemostasis system when combined with the fibrinolytic system and platelets. The coagulation cascade activation involves primary and secondary hemostasis, often coinciding with the activation of inflammatory mechanisms.^27^ Primary hemostasis involves platelet activation and aggregation, leading to fibrin formation by thrombin. This event triggers an acute inflammatory response to control tissue damage, halt blood loss, and prevent microbial infection. During secondary hemostasis, plasmin dissolves fibrin and reparative inflammatory cells, collectively working to remodel and repair damaged tissue.^28^ Under normal conditions, a tightly regulated hemostasis system poses minimal risk of complications or failed responses. However, dysregulation can lead to overactive platelet responses, abnormal fibrin formation, and impaired fibrinolysis, creating an environment conducive to cardiovascular events. Furthermore, the intercommunication between the complement and coagulation systems can amplify inflammatory processes within the vascular system, heightening the risk of cardiovascular events.

Like the complement and coagulation system, lipid metabolism is also related to comorbidities with high mortality rates. Lipid metabolism refers to the biochemical processes involving the organism's production, transport, storage, and utilization of lipids. Dysregulation of this process can result in conditions such as elevated lipid levels (hyperlipidemia), the development of atherosclerosis, and various cardiovascular diseases.^29^ Atherosclerosis is a condition driven by lipoproteins, resulting in the development of plaques along the arterial tree through processes such as inflammation and necrosis. Increased concentrations of lipoproteins containing apolipoprotein B, mainly low-density lipoprotein (LDL), are often a primary factor in the independent development of atherosclerosis. Furthermore, the disease often develops at lower levels of LDL in combination with additional risk factors such as smoking, hypertension, and diabetes mellitus.^30^ Literature also shows that lipid metabolic pathways undergo evident changes in respiratory illnesses such as chronic obstructive pulmonary disease. The lipid modifications enable lung tissue to activate anabolic pathways that initiate the synthesis of active molecules directly involved in inflammation. There is evidence suggesting that obesity is linked to decreased lung function and heightened morbidity in individuals with chronic obstructive pulmonary disease.^31^ Also, lipid metabolism is traditionally considered important in the risk factors of ischemic stroke, encompassing elevated levels of total cholesterol, triglycerides, LDL cholesterol, and reduced high-density lipoprotein cholesterol levels. Additionally, managing hyperlipidemia is essential not only for reducing the risk of cardiovascular disease but also for preventing strokes.^32^ In individuals diagnosed with BD, a prevalent occurrence of these comorbidities may contribute to an elevated mortality rate. Several of these comorbidities are a consequence of a sedentary lifestyle, tobacco use, heightened alcohol consumption, and chronic exposure to stress.^5^ A comprehensive understanding of the molecular fundaments involving lipid metabolism, complement activation, and coagulation cascade abnormalities establishes an essential link between cellular mechanisms and comorbidities clinical manifestations in BD. Thus, complement and coagulation systems and lipid metabolism may be potential mediators of BD's early onset of medical comorbidities. These findings suggest that collaborative care interventions focusing on the treatment of these comorbidities can significantly decrease early death among individuals with BD.^3^

Furthermore, we cannot rule out the possible effect of psychotropic medications on our results since all patients in this study were under pharmacological treatment. Mood stabilizers, as well as many atypical antipsychotics commonly used for the treatment of BD, can promote weight gain and, thus, the entire cascade of metabolic effects that lead to long-term cardiovascular complications. Nevertheless, it's crucial to acknowledge that, despite the apparent heightened risk of cardiovascular diseases linked to numerous psychotropic medications, there is no conclusive evidence that these medications autonomously amplify the risk of cardiovascular disease in individuals with BD. Some evidence opposes this hypothesis, suggesting that enhanced symptom control might facilitate improved health-related behaviors, increased utilization of health services, and, indirectly, better physical health.^5^

Understanding the molecular pathways and processes identified in the study opens avenues for targeted interventions, including molecular mechanisms underlying BD and its associated medical comorbidities. Specifically, targeting proteins associated with lipid metabolism, the complement system, and the coagulation cascade could mitigate the systemic alterations observed in patients with BD. Early identification of systemic alterations enables the development of preventive strategies, reducing the risk of medical comorbidities and ultimately reducing mortality rates. Pharmaceutical interventions or lifestyle modifications to modulate these pathways could be explored to prevent or alleviate medical comorbidities. Ultimately, our findings underscore the importance of integrated care models that address the mental and physical health needs of patients with BD. Collaborative care approaches involving mental health professionals, primary care physicians, and specialists in relevant medical fields could ensure holistic management of BD and its associated comorbidities, improving patient outcomes. By adopting personalized, mechanism-based interventions and integrating mental and physical health care, clinicians can strive towards more effective and integral care for individuals with BD.

Some limitations should be considered when interpreting the results of this study. The sample size was relatively small, so the type I error cannot be ruled out. Convenience sampling was used, which may have limited the generalizability of the results. All patients were on pharmacological treatment. The polypharmacy introduces additional complexities that warrant thorough analysis since the pharmacological agents can directly influence the investigated biological responses and generate synergistic or antagonistic effects in signaling pathways and observed outcomes. Also, individual variability in treatment response is a significant factor, making it challenging to draw definitive conclusions about the associations between identified proteins and comorbid health conditions. Overall, the medications taken by the patients are likely to have multifaceted effects on the results, influencing the observed biological responses. Therefore, they must be considered limiting factors in interpreting the study's findings. Also, the present study employed a cross-sectional design, which inherently limits our ability to establish causal relationships between the observed variables. Some factors, like diet and infections, may have short-term effects on the technique used, introducing noise into the data and potentially leading to biased results. Future research utilizing a longitudinal design with repeated measurements would be beneficial in strengthening the causal inferences drawn from this study. Algorithms and analysis methods can significantly influence the results. Different algorithms may yield distinct interpretations from the same datasets, and the biological interpretation of results obtained through bioinformatic approaches can be challenging. Due to this, we partnered with an experienced bioinformatician to work with the data. Furthermore, a proteomic profile was obtained from a blood sample, potentially not fully capturing the complex molecular changes associated with BD throughout the brain.

## Conclusion

In summary, despite the exploratory analysis, this is the first study to identify protein profile, using LC-MS/MS, within the peripheral blood across various stages of BD. Our findings underscore specific proteins primarily linked to lipid metabolism, the complement system, and the coagulation cascade. These findings may represent systemic alterations that occur with the progression of the illness and a possible link between BD and medical comorbidities. This comprehensive understanding provides valuable insights for targeted interventions, addressing mental health aspects and mechanisms driving comorbidities in BD. While our findings offer valuable insights, several recommendations for future research directions emerge: a) conduct larger-scale studies with increased sample sizes, including a more diverse sample population, to enhance statistical power and improve the generalizability of findings; b) implement longitudinal study designs to assess how changes in protein expression correlate with clinical outcomes following pharmacological or psychosocial interventions; c) utilize multi-omics approaches, integrating data from genomics, transcriptomics, proteomics, and metabolomics, to investigate the molecular pathways involved in BD comprehensively; d) conduct functional studies to validate the biological relevance of identified proteins and elucidate their roles in disease pathogenesis.

## Supplementary Material


