# Supplementary material for: Proteomic insights into biology of bipolar disorder: implications for health complexity and mortality
Source: Trends Psychiatry Psychother. 2025 Apr 17;47:e20240820. doi: 10.47626/2237-6089-2024-0820 (PMC12904327; doi:10.47626/2237-6089-2024-0820)
Supplement: Supplementary file 1 [file 2238-0019-trends-47-e20240820-suppl01.pdf]

http://doi.org/10.47626/2237-6089-2024-0820

Supplementary Figure S1 - Protein intersection in comparison groups. Venn diagram displaying proteins identified in bipolar disorder (BD), with poor functioning (BD-) and BD with high functioning (BD+) comparison groups. HC = healthy controls.

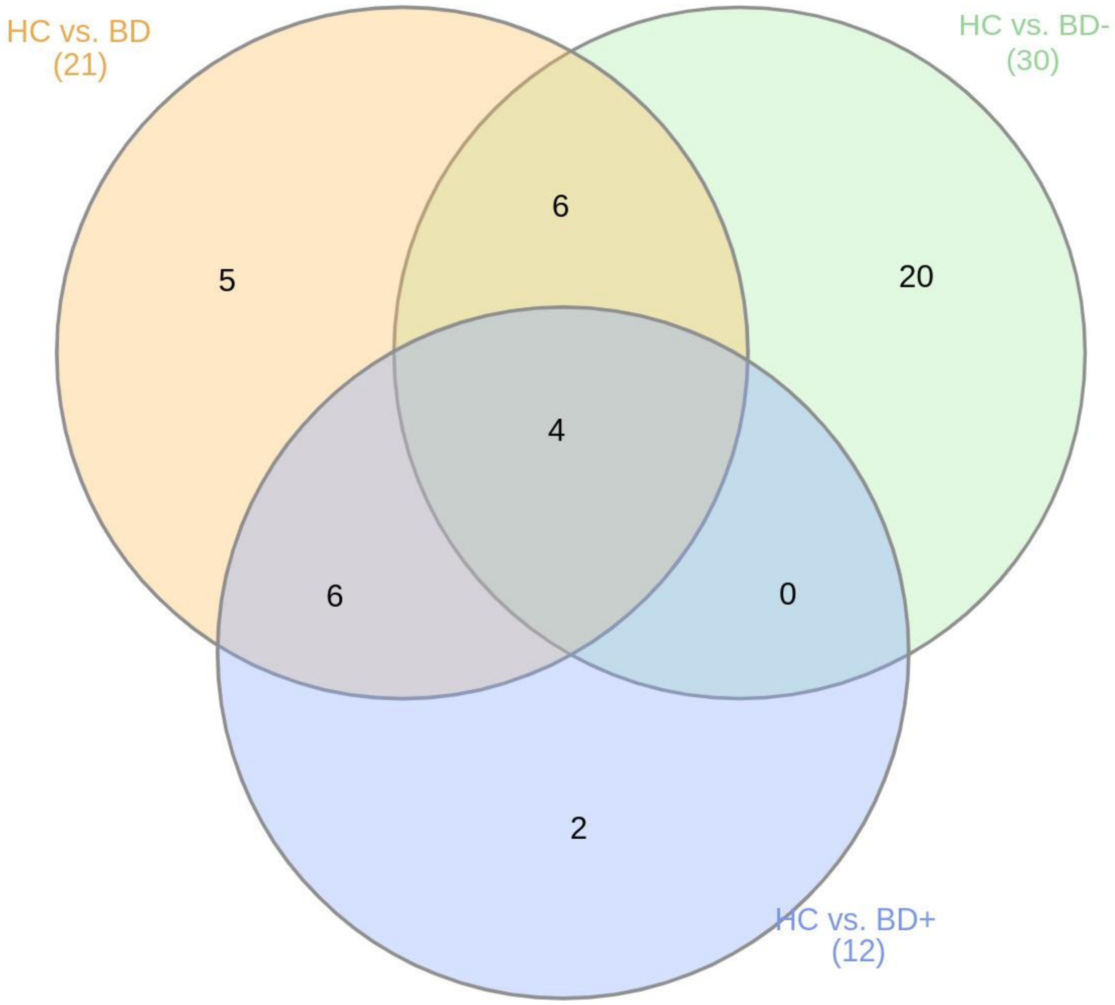

http://doi.org/10.47626/2237-6089-2024-0820

Supplementary Table S1 - Proteins identified in the blue module. A list of proteins found within the blue module and relevant Kyoto Encyclopedia of Genes and Genomes (KEGG) pathways

Gene names	Protein IDs	Protein names	KEGG pathway
CD163	Q86VB7	Scavenger receptor cysteine-rich type 1 protein M130	
CFP	P27918	Properdin	
ATRN	O75882	Attractin	
FCN3	O75636	Ficolin 3	
C8B	P07358	Complement component C8 beta chain	
HABP2	Q14520	Hyaluronan-binding protein 2	
MASP2	O00187	Mannan-binding lectin serine protease 2	
C8A	P07357	Complement component C8 alpha chain	
CSF1R	P07333	Macrophage colony-stimulating factor 1	
LDHB	P07195;	L-lactate dehydrogenase B chain	
MASP1	P48740	Mannan-binding lectin serine protease 1	
OLFM1	Q99784	Noelin	
APOC2	P02655	Apolipoprotein C-II	
BTD	P43251	Biotinidase	
PCYOX1	Q9UHG3	Prenylcysteine oxidase 1	
AFM	P43652	Afamin	
DCD	P81605	Dermcidin	
C4BPB	P20851	C4b-binding protein beta chain	Complement and coagulation cascades
APOM	O95445	Apolipoprotein M	
HSPA5	P11021	Endoplasmic reticulum chaperone BiP	
APOC4	P55056	Apolipoprotein C-IV	
PTPRJ	Q12913	Receptor-type tyrosine-protein phosphatase eta	
CTBS	Q01459	Di-N-acetylchitobiase	
SERPINA6	P08185	Corticosteroid-binding globulin	
APOC1	P02654	Apolipoprotein C-I	
APOB	P04114	Apolipoprotein B-100	
CLU	P10909	Clusterin	
APOC3	P02656	Apolipoprotein C-III	
LGALS3BP	Q08380	Galectin-3-binding protein	
DPP4	P27487	Dipeptidyl peptidase 4	
CD5L	O43866	CD5 antigen-like	
IL1RAP	Q9NPH3	Interleukin-1 receptor accessory protein	
COMP	G3XAP6	Cartilage oligomeric matrix protein	
GPLD1	P80108	Phosphatidylinositol-glycan-specific phospholipase D	
QSOX1	O00391	Sulfhydryl oxidase 1	
THBS4	P35443	Thrombospondin-4	
